# Prognostic Value of Preoperative Albumin-to-Alkaline Phosphatase Ratio for Survival in Colorectal Cancer Patients Undergoing Surgery

**DOI:** 10.3390/jcm14030901

**Published:** 2025-01-29

**Authors:** Hacı Arak, Ercan Gumusburun, Mustafa Seyyar, Havva Yesil Cinkir

**Affiliations:** 1 Department of Medical Oncology, Gaziantep City Hospital, TR-27010 Gaziantep, Turkey; mustafaseyyar27@hotmail.com; 2 Department of Internal Medicine, Faculty of Medicine, Şahinbey Training and Research Hospital, Gaziantep University, TR-27470 Gaziantep, Turkey; ercangmsbrn@gmail.com; 3 Department of Medical Oncology, Sahinbey Training and Research Hospital, Gaziantep University, TR-27470 Gaziantep, Turkey; drhavva1982@gmail.com

**Keywords:** albumin–alkaline phosphatase ratio, neutrophil-to-lymphocyte ratio, colorectal cancer, prognostic value

## Abstract

**Background and Objectives:** This study aimed to evaluate the prognostic significance of the pre-treatment albumin-to-alkaline phosphatase ratio (AAPR) in early-stage colorectal cancer patients and to compare it with the neutrophil-to-lymphocyte ratio (NLR) and platelet-to-lymphocyte ratio (PLR) within the same patient cohort. **Materials and Methods:** This retrospective study included 540 patients who were followed up after a diagnosis of early-stage colorectal cancer and whose albumin (ALB), alkaline phosphatase (ALP), neutrophil, platelet, and lymphocyte values were measured before treatment. **Results:** In the receiver operating characteristic (ROC) curve analysis for overall survival (OS), the AAPR index Area Under Curve (AUC):0.560, (*p* = 0.018), NLR index (*p* = 0.079), and PLR index (*p* = 0.692) were evaluated. In the ROC analysis for OS, a cut-off value of the AAPR index of ≤0.423 was found. In the AAPR-low group, the relapse and death rates were higher than in the AAPR-high group (*p* = 0.004 and *p* = 0.001, respectively). As the AAPR index decreased, the NLR and PLR indexes increased (*p* = 0.027 and *p* = 0.003, respectively). Median disease-free survival (DFS) was worse in the AAPR-low group (128 versus 156) months (*p* = 0.015). The AAPR index significantly affected OS with hazard ratio (HR):0.42 (95%CI, 0.18–0.97) (*p* = 0.044). At 5 years, 68% of the patients in the AAPR-low group and 79% of the patients in the AAPR-high group were alive (*p* = 0.005). In a multivariate analysis, low AAPR, patient age at diagnosis, TNM stage, and recurrence status were independent factors affecting OS (*p* = 0.022, *p* < 0.001, *p* = 0.002, and *p* < 0.001, respectively). **Conclusions**: In early-stage colorectal cancer patients, the OS was worse in the AAPR-low group than in the AAPR-high group. The AAPR index demonstrated significant prognostic value for OS compared to the NLR and PLR in the same patient cohort.

## 1. Introduction

Colorectal cancer is the third leading cause of cancer and second most common cause of cancer-related deaths worldwide. In recent years, colorectal cancer has started to appear at earlier ages, and its incidence is increasing [1]. Approximately 80% of patients are diagnosed at local stage. However, during follow-up, approximately half of the patients develop recurrence, and the disease progresses to the metastatic stage. Therefore, clinical and pathological risk factors that can predict recurrence in colorectal cancer patients undergoing surgery are important. Clinical and pathological factors, such as patient performance status, patient age, tumor location, tumor stage, lymph node stage, tumor deposits, lymphovascular invasion(LVI), perineural invasion(PNI), microsatellite instability status, and tumor grade are crucial for treatment and follow-up in clinical practice [2].

Even in patients with local colorectal disease at the same stage, disease-free survival (DFS) and overall survival (OS) may differ. Biomarkers that predict disease recurrence can help identify high-risk patients who may benefit from adjuvant therapy or treatment escalation. In recent years, circulating tumor DNA and circulating tumor cells have been investigated post-surgery in colorectal cancer patients, similar to research in other cancer types [3]. However, these methods are not yet recommended in treatment guidelines and are expensive. Therefore, there is a need for inexpensive and easily accessible biomarkers to predict postoperative DFS and OS in resectable colorectal cancer patients.

Recent studies have shown that inflammation plays a critical role in all processes of cancer [4]. Indices derived from blood test parameters such as neutrophils, lymphocytes, platelets, and monocytes are being investigated for their relationship with DFS, PFS, and OS across many cancer subtypes [5]. Zou et al. found that survival outcomes in colorectal cancer patients who have undergone surgery worsen as the neutrophil-to-lymphocyte ratio (NLR) and platelet-to-lymphocyte ratio (PLR) increase [6]. The prognostic significance of NLR and PLR has been explored in many cancers, including colorectal cancer. However, there is a need for indices with better prognostic value that are more useful in clinical practice.

Albumin (ALB), produced by the liver, reflects nutritional and inflammation status as it is a negative-phase reactant, indirectly influencing immune function. In cancer patients, decreased ALB levels are associated with systemic inflammation and malnutrition [7]. Alkaline phosphatase (ALP) is a group of isoenzyme complexes found in the liver, bones, and kidneys. ALP catalyzes the removal of phosphate groups from nucleic acids. High ALP levels may indicate bone disease, cholestasis, systemic inflammation, tumor invasion, and metastasis potential. Pre-treatment ALP elevation was associated with liver and bone metastasis in cancer patients. Pre-treatment ALP elevation was associated with poor survival, low albumin levels, and serum carcinoembryonic antigen (CEA) elevation in colorectal cancer patients [8]. Anthony et al. first demonstrated that the albumin-to-alkaline phosphatase ratio (AAPR) is an important prognostic predictor of hepatocellular carcinoma [9]. A low AAPR has been associated with poorer DFS and OS in patients who have undergone surgery for non-small cell lung and gastric cancer [10,11]. In the literature on colorectal cancer, a low preoperative AAPR index has been linked to poor postoperative survival in a study of 221 patients who underwent laparoscopic surgery [12].

The aim of this study was to comprehensively investigate the prognostic significance of the AAPR index for DFS and OS in patients who underwent surgery for stage 1–3 colorectal cancer and to compare the prognostic significance of the AAPR, NLR, and PLR indices in the same patient group.

## 2. Materials and Methods

### 2.1. Study Design

This retrospective, single-center, blood result-based study was conducted in accordance with the principles of the Declaration of Helsinki. The requirement for informed consent was waived owing to the retrospective nature of this study. No funding was received for this study. This study was approved by the Gaziantep University Faculty of Medicine Ethics Committee (approval no.2023/280).

A list of 1938 patients diagnosed with colorectal disease in our center in the last 10 years was obtained. Repeat diagnoses were removed. Oncology patients are followed up with a manual file and electronic system in our center. The manual file of each patient was examined in detail, patients who met the exclusion criteria were excluded, and the data of the appropriate patients were recorded in the SPSS program. The inclusion criteria were patients aged ≥18 years with a pathologically confirmed colon adenocarcinoma diagnosis, those undergoing surgery for localized stage colon cancer with ALB, ALP, and hemogram results available within 7 days before treatment, and those whose data were accessible. Patients with liver cirrhosis, decompensated heart failure, known secondary cancer, metastatic colon cancer, and those for whom follow-up and treatment data were not available were excluded. Patients who died in the first 1 month postoperatively owing to surgical complications were excluded. Between January 2012 and January 2022, 540 patients diagnosed with colorectal cancer were eligible for this study.

### 2.2. Variables and Definitions

Age at diagnosis, sex, localization, lymphovascular invasion (LVI); tumor deposit, differentiation status, tumor stage, lymph node stage, preoperative ALB, ALP, and hemogram (neutrophils, lymphocytes, and platelets) examination results, tumor markers, adjuvant chemotherapy agents received by the patients, recurrence dates, and last follow-up or death dates of the patients were retrospectively scanned from patient files or electronic systems. The patients were followed up using contrast-enhanced computed tomography to determine the recurrence status, that is, the presence of liver, lung, non-regional lymph node, or peritoneal metastases. The NLR was defined as the number of neutrophils divided by the number of lymphocytes in the pre-treatment hemogram. The PLR was defined as the number of platelets divided by the number of lymphocytes. AAPR was calculated as the ratio of serum ALB to serum ALP levels (the units for ALB and ALP are g/L and U/L, respectively). DFS was defined as the time from the date of surgery to the date of recurrence, last follow-up, or death, whichever occurred first. OS was defined as the time from the date of diagnosis to the date of the last follow-up or death.

### 2.3. Statistical Analysis

The Shapiro–Wilk test analyzed the normal distribution of the numerical data. The chi-squared test evaluated the relationships between categorical variables, while correlation coefficients were used for numerical variables. Categorical data are presented as percentages, and continuous variables are expressed as median (minimum–maximum or interquartile range [IQR]). Student’s *t*-test compared variables with normal distribution in the two groups, and the Mann–Whitney U test compared variables that were not normally distributed in the two groups. One-way ANOVA determined the mean age at diagnosis according to the tumor localization. A receiver operating characteristic (ROC) curve analysis was performed to assess the optimal cutoff values for AAPR, NLR, and PLR separately for patients in different cancer stages. Patients below the cut-off value were included in the “Low group”, and those above the cut-off value were included in the “High group”. The Kaplan–Meier method and log-rank test analyzed the DFS and OS of the different groups. Variables affecting OS or DFS were evaluated in both univariate and multivariate analyses using the Cox proportional hazards regression model. SPSS (version 22.0) was employed for all the analyses. Statistical significance was set at *p* <0.05.

## 3. Results

### 3.1. Patient Characteristics

A total of 540 patients who underwent surgery for colorectal cancer were eligible for inclusion. The median age of the patients was 59 (IQR, 50–67) years. Of the patients, 302 (56%) were male, 353 (65%) had colon cancer, 187 (35%) had rectal cancer, 253 (47%) had stage-2 cancer, 219 (41%) had recurrence, and 189 (35%) died. Patient age at diagnosis, sex, performance score (PS), adjuvant chemotherapy status, median ALB and ALP values, LVI, PNI, differentiation grade, tumor stage, lymph node stage, TNM (Tumor, Nodes, Metastases) stage, recurrence status, and rates of living and dead patients are summarized in Table 1. The median age at diagnosis in right colon cancer patients was 58 years, and the median age at diagnosis in left colon cancer patients was 61 years (*p* = 0.025).

In the ROC analysis for DFS, the AAPR index (AUC: 0.548, *p* = 0.058), NLR (AUC: 0.525, *p* = 0.328), and PLR indices (AUC: 0.522, *p* = 0.392) did not reach statistical significance. In the ROC analysis for DFS, pre-treatment CEA level (AUC:0.569, *p*= 0.007) and platelet count (AUC:0.583, *p* = 0.001) were significant. Additionally, in the ROC analysis for DFS, the AAPR index AUC:0.582 (95%CI,0.211–0.658) (*p* = 0.028) was significant in stage-2 patients. There were no significant differences in the median AAPR, NLR, and PLR values between the 353 (65%) colon cancer patients and 187 (35%) rectal cancer patients (*p* = 0.498, *p* = 0.070, and *p* = 0.081, respectively). As the NLR increased, the PLR index also increased, indicating a positive correlation (Pearson correlation R: 0.714, *p*< 0.001). There was a negative correlation between AAPR, NLR, and PLR (R: −0.095, *p* = 0.027 and R: −0.127, *p* = 0.003, respectively). There was a negative correlation between the AAPR index and preoperative CEA and CA19.9(cancer antigen 19-9) serum levels, but it was not significant (*p* = 0.232, *p* = 0.110, respectively). As tumor differentiation progressed from good to worse, the number of patients receiving adjuvant chemotherapy increased (*p*< 0.001).

**Figure 1:**
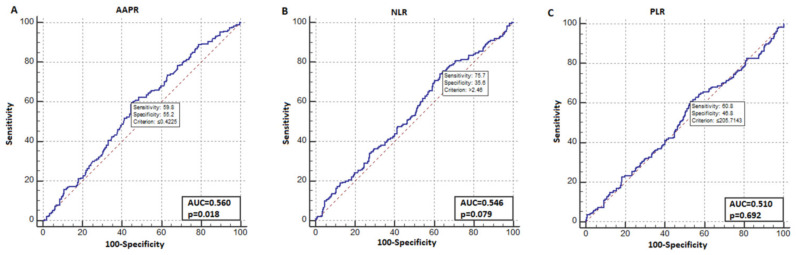
Receiver operating characteristics (ROC) analysis for overall survival. (**A**) Albumin–alkaline phosphatase ratio (AAPR), (**B**) neutrophil–lymphocyte ratio (NLR), (**C**) platelet–lymphocyte ratio (PLR).

In the ROC analysis for OS, the AAPR index had an AUC of 0.560 (95%CI, 0.517–0.603), (*p* = 0.018), the NLR index had an AUC of 0.546 (*p* = 0.079), and the PLR index had an AUC of 0.510 (*p* = 0.692) (Figure 1). In the ROC analysis for OS, the AAPR index was found to have a significant optimal cut-off value of ≤0.423. The sensitivity and specificity of this cutoff value were 60% and 55%, respectively. Patients with AAPR values ≤0.423 were included in the AAPR-low group, and those with AAPR values >0.423 were included in the AAPR-high group. The number of patients in the AAPR-low and AAPR-high groups was equal, with 270 in each group. The median AAPR value was 0.329 (IQR: 0.271–0.371) in the AAPR-low group and 0.528 (IQR: 0.475–0.641) in the AAPR-high group (*p*< 0.001). The baseline characteristics and distributions of the two patient groups are summarized in Table 1. In the AAPR-low group, the number of patients with recurrence and the number of patients who died were higher than in the AAPR-high group (*p* = 0.004 and *p* = 0.001, respectively). As expected, the median ALB level was lower, and the median ALP level was higher in the AAPR-low group than in the AAPR-high group (*p* = 0.001 for both).

### 3.2. Survival Analysis

**Figure 2:**
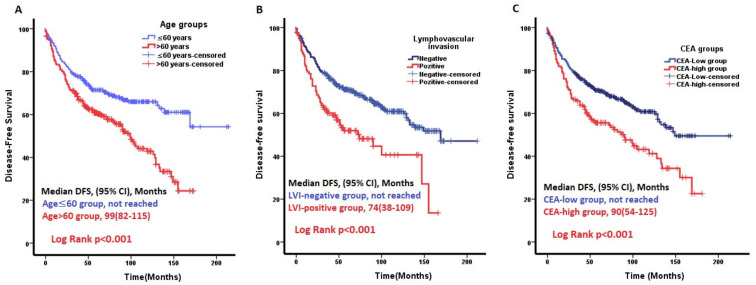
Factors independently affecting disease-free survival (DFS) in multivariable analysis. (**A**) The median DFS was superior in patients aged ≤60 years at diagnosis than in those aged ≥60 years (*p* < 0.001). (**B**) Lymphovascular invasion (LVI) positive patients had worse DFS than that of the LVI negative patients (*p* < 0.001). (**C**) In the ROC analysis of the patients’ pre-treatment CEA levels for DFS, a cut-off value of 2.8 was found. The DFS rate of the patients in the CEA-low group was better than that of the CEA-high group.

In our study, the median DFS of the study cohort was 133 months (95%CI, 113–152). At 5 years, 66% of the patients were disease-free. A total of 368 (68%) patients received adjuvant treatment for colon or rectal cancer. The treatments received were as follows: 166 (45%) patients received capecitabine or 5-fluorouracil infusion, and 202 (55%) patients received oxaliplatin plus capecitabine or oxaliplatin plus 5-fluorouracil infusion. The median DFS in AAPR-low group patients was 128 (95%CI, 100–156) months, and that in the AAPR-high group patients was 149 (95%CI, 126–171) months (*p* = 0.015). In the univariate analysis, age at diagnosis, LVI, PNI, TNM stage, CEA, CA19-9, platelet count, ALB values, adjuvant chemotherapy, tumor deposit, T stage, N stage, and AAPR group were found to be significantly associated with DFS. Since the N and T stages are components of the TNM stage, the TNM stage was included in the multivariable analysis. In the multivariate analysis, age at diagnosis, LVI, PNI, CEA levels, and pre-treatment ALB levels were significantly associated with DFS (*p* < 0.001, *p* = 0.028, *p* = 0.044, *p* = 0.012, and *p* = 0.042, respectively). Kaplan–Meier plots of age at diagnosis, LVI, and pre-treatment CEA levels, which were significant parameters for DFS in multivariate analysis, are shown in Figure 2.

**Figure 3:**
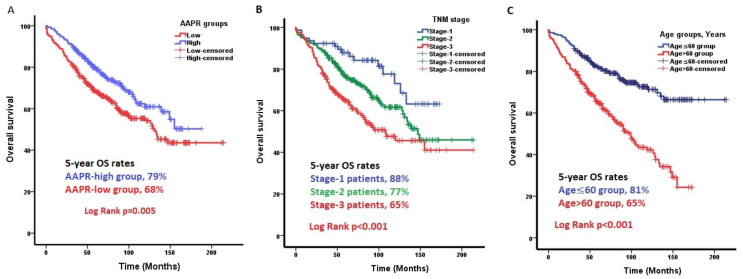
Factors that independently affected overall survival (OS) in multivariable analysis. (**A**) OS of patients in the AAPR-low group was worse than that in the AAPR-high group (*p* = 0.005). (**B**) According to the TNM stage, the OS of the patients worsened as the stage increased (*p* < 0.001). (**C**) OS was superior in patients aged ≤60 at diagnosis than in those aged ≥60 (*p* < 0.001).

Median OS was not reached in the study cohort. At 5 years, 74% of patients were alive. In the univariate analysis, the AAPR index significantly affected OS with hazard ratio (HR) of 0.42 (95%CI, 0.18–0.97, *p* = 0.044). At 5 years, 68% of the patients in the AAPR-low group and 79% of the patients in the AAPR-high group were alive (*p* = 0.005). In the univariate analysis of OS, the NLR index had an HR of 1.023 (95%CI, 1.000–1.046), (*p* = 0.053), and the PLR index had an HR of 1.000 (95%CI, 0.999–1.001), (*p* = 0.629). Clinical, pathological, and biochemical factors that may affect OS were analyzed using univariate Cox regression. Significant factors were included in the multiple Cox regression (Table 2). In multivariate analysis, low AAPR, patient age at diagnosis, TNM stage, and recurrence status were independent factors affecting OS (*p* = 0.022, *p* < 0.001, *p* = 0.002, and *p* < 0.001, respectively). Kaplan–Meier plots of the pre-treatment AAPR index, TNM stage, and age at diagnosis, which were significant parameters for OS in multivariate analysis, are shown in Figure 3.

## 4. Discussion

In this retrospective study, we investigated the prognostic significance of preoperative AAPR in a large cohort of early-stage colorectal cancer patients. The overall survival of patients in the AAPR-low group was worse than that of patients in the AAPR-high group. While the prognostic value of the NLR and PLR indices was not significant in our study, the preoperative AAPR index was found to be an independent parameter of OS in early-stage colorectal cancer patients.

Hypoalbuminemia may be a sign of liver synthesis dysfunction, nephrotic syndrome, malnutrition, and inflammation. Preoperative ALB has been shown to be associated with increased morbidity and surgical complications in colorectal cancer patients [13]. ALP is a hydrolytic enzyme complex involved in dephosphorylation and transphosphorylation. ALP is frequently elevated in liver disease, bone metastasis, kidney disease, and biliary tract cholestasis. In colorectal cancer patients, preoperative ALP levels are associated with advanced disease stages and poor survival [8]. ALP and ALB levels are affected by various conditions and factors; therefore, the AAPR is believed to be a more objective index, and its prognostic significance has been investigated in some cancers. In patients who underwent surgery for gastric cancer [10] and non-small cell cancer [11], a low pre-treatment AAPR was found to be associated with poor DFS and OS. Furthermore, when the AAPR index was combined with the TNM stage in the study cohort, AAPR-TNM staging predicted OS more accurately than TNM staging [11]. Long et al. revealed that lymph node metastasis and the pre-treatment AAPR index are independent risk factors affecting OS in non-metastatic breast cancer patients [14]. Jiang et al. conducted a multivariable analysis in their study of 221 patients who underwent laparoscopic colectomy and reported that the preoperative AAPR index, age, tumor grade, TNM stage, and CEA level are significant predictors of OS [12]. Similar to the aforementioned studies, in our study, multivariable analysis revealed that the pre-treatment AAPR index was one of the independent factors influencing OS. Additionally, the rate of patients developing recurrence and the rate of patients dying in the AAPR-low group were significantly higher than those in the AAPR-high group. Jiang et al. reported a 5-year survival rate of 78% in 221 patients who underwent only laparoscopic surgery [12]. Our study included 540 patients who underwent frequent open surgeries and found 5-year survival rate of 74%. In addition, the importance of NLR and PLR, together with AAPR, was investigated in our study. While the AAPR index was significant for OS, the NLR and PNL indices were not. NLR and PLR indicated acute inflammation but did not predict OS in our patient cohort. However, the AAPR index was considered a significant factor for OS because it closely reflects malnutrition, chronic inflammation, and immune status.

The prognostic effect of the AAPR index on DFS across various surgically treated cancers is evident in the subgroup analysis of the studies. Wang et al., using multivariate analysis, found that the AAPR index is a significant predictor of DFS in gastric cancer, along with the TNM stage [10]. In a study by Zhang et al., the AAPR index predicted DFS in both adenocarcinoma and squamous cell lung cancer patients [11]. In the study by Long et al. of non-metastatic breast cancer patients and the study by Jiang et al. of non-metastatic colorectal cancer patients, median survival was not reached, and the effect of the AAPR index on DFS was not analyzed [12,14]. In the aforementioned study on gastric cancer patients, the median DFS in the AAPR-low group was 15 months, and, in non-small-cell lung cancer studies, the median DFS in the AAPR-low group was 29 months. Whereas in the present study, the median DFS of the study cohort was 133 months. The AAPR index for DFS was not significant in the ROC analysis because the DFS in gastric cancer and non-small cell lung cancer patients is shorter than that in non-metastatic colorectal cancer patients. The median DFS of patients in our study was approximately 11 years; therefore, the prognostic effect of the AAPR index on DFS in our study was not significant. In addition, 139 (26%) of 219 (40.6%) patients with recurrence had radiological recurrence. Eighty (14.8%) of 189 (35%) patients had died of non-cancer reasons without radiological recurrence. The AAPR index reflects malnutrition, nephropathy, and liver functionin addition to cancer, giving it prognostic value for patient mortality. However, as ALB and ALP, which are components of the AAPR index, are not directly produced or reduced by tumors, they may not serve as prognostic factors for recurrence. In our study for DFS in the multivariate analysis, age at diagnosis, pre-treatment ALB level, and tumor-related parameters such as LVI, PNI, and CEA levels were significant. There are some reasons why PNI, LVI, CEA, age at diagnosis, and ALB levels are significant parameters in the multivariable analysis for DFS. Tumor grade, LVI, and PNI are among the risk factors determining the adjuvant treatment effect in early-stage colorectal cancer patients and are parameters associated with recurrence. We think that the reason age is significant in the Cox regression model is that non-cancer causes of death increase with age, thus affecting DFS. CEA is a marker frequently secreted by tumors, and high CEA has been associated with postoperative recurrence in other studies [15]. The low ALB level may have been significant for DFS because ALB levels reflect malnutrition, inflammation, and immune status better than ALP levels.

In previous studies, the prognostic significance of the systemic inflammation indices NLR and PLR for DFS and OS in colorectal has been controversial. Lee et al. reported that the NLR index, a serum inflammatory marker, has no significant prognostic significance for OS and DFS in a multivariate analysis of 1590 colorectal cancer patients [16]. Cho et al. reported that the prognostic significance of the pre-treatment NLR index for OS in multivariable analysis in 623 stage 2–3 colorectal cancer patients was significant (*p* = 0.041) [17]. One study found that both NLR and PLR have prognostic significance for survival in colon cancer patients [6], whereas another study reported that neither NLR nor PLR was significant for survival in stage-3 colon cancer patients [18]. In our study, the prognostic significance of NLR and PLR for DFS and OS was not significant. NLR and PLR indices indirectly reflect inflammation owing to the presence of tumors or other factors. Cancer prognosis is related to the biological characteristics of the tumor and the body’s response [19]. The inconsistent results in previous studies may be attributed to differences in the societies and geographies where they were conducted, variations in sample sizes, and the inclusion of patients at different cancer stages. However, in our study, although the NLR and PLR indices were not significant for OS in the same patient group, the AAPR index was significant. NLR and PLR are mainly related to inflammation, while ALB in the AAPR component is associated with both inflammation and malnutrition. However, for indices predicting tumor prognosis, combining markers that indicate body inflammation, patient malnutrition, and tumor burden may provide more accurate information [20]. Additionally, although not currently standard practice owing to its availability and cost, circulating tumor DNA can be used to detect postoperative molecular residual disease, monitor adjuvant chemotherapy efficacy, and identify early recurrence during surveillance in early-stage colorectal cancer patients [21]. To the best of our knowledge, our study is the first to compare AAPR, NLR, and PLR indices in the same patient cohort who underwent surgery for colorectal cancer.

There is no standard cut-off value for AAPR, NLR, and PLR indexes in colorectal cancer, as in other cancers. However, similar cut-off values obtained for these indexes in different studies may increase their applicability in clinical practice. In our study, the AAPR was found to be 0.423. In another early-stage colorectal cancer study, the cut-off was found to be 0.495 [12], and in a meta-analysis conducted for other cancers, the cut-off was between 0.35 and 0.64 [22]. In our study, the cut-off value for NLR in ROC analysis was 2.46, and the median NLR value was 3.39 (IQR, 2.19–5.49). Chiang et al. found the 3.0 cut-off value in ROC analysis of 3857 patients with operated colorectal cancer [23]. Our study found the cut-off value for PLR was 250, with a median of 189 (IQR, 133–287). In the review investigating the prognostic significance of PLR in colorectal cancer, the PLR index was prognostic for OS but not significant for DFS, and the cut-off was seen to be in the range of 150–300 [24]. There are reasons for the different cut-off values of the indexes in colorectal cancer patients, such as different populations, disease stages, and preoperative or postoperative examinations.

Our study had certain limitations. It was a retrospective, single-center study with no standard cut-off values for AAPR, NLR, or PLR indices in colorectal cancer. Selective biases are inherent in retrospective studies. The lack of a standard cut-off value may limit the use of blood-based indices in routine practice. External validation studies are needed to determine the standard cut-off values of these indices. However, our study comprehensively investigated the prognostic significance of the AAPR index in a large cohort of patients. While the AAPR index demonstrated prognostic significance in the same patient cohort, the NLR and PLR indices did not. Future studies should investigate the correlation between the AAPR index and circulating tumor DNA, as well as the impact of adding AAPR to TNM staging for prognostic assessment.

In conclusion, the AAPR is a simple, inexpensive, noninvasive, and accessible blood biomarker-based index. In patients who underwent surgery for colorectal cancer, the AAPR index showed significant prognostic value for OS, while the NLR and PLR indices did not. Patients with low AAPR scores had worse OS than those with AAPR-high scores. The lack of significance of AAPR for DFS may be due to the occurrence of non-cancer-related deaths in patients without recurrence and a longer median DFS. Among the AAPR components, ALB significantly predicts DFS better than ALP. A low AAPR index was considered an independent parameter for OS, such as TNM stage, age at diagnosis, and development of recurrence. The proportion of patients who developed recurrence and died was significantly higher in the AAPR-low group. The lack of a standard cut-off value may limit the use of blood-based indices in routine practice. Future studies should investigate the correlation between the AAPR index and circulating tumor DNA and the contribution of adding the AAPR index to TNM staging in multicenter randomized prospective trials.

## Figures and Tables

**Table 1:** Distribution of baseline characteristics between patients in the AAPR-low and AAPR-high groups.

Variables		All, (*n*:540), *n* (%)	AAPR≤0.423 (*n*:270), *n* (%)	AAPR > 0.423 (*n*:270), *n* (%)	*p* * Value
Age (median [min–max]) years.		59 (20–91)	60 (23–86)	58 (20–91)	0.231
Sex	Male	302 (55.9)	145 (53.7)	157 (58.1)	0.298
	Female	238 (44.1)	125 (46.3)	113 (41.9)	
Performance score (PS)	PS-0	96 (17.8)	39 (14.4)	57 (21.1)	0.066
	PS-1	378 (70)	197 (73)	181 (67)	
	PS-2	36 (6.7)	15 (5.6)	21 (7.8)	
	PS-3	30 (5.6)	19 (7)	11 (4.1)	
Location of the tumor	Right	167 (30.9)	84 (31.1)	83 (30.7)	0.842
	Left	186 (34.4)	90 (33.3)	96 (35.6)	
	Rectum	187 (34.6)	96 (35.6)	91 (33.7)	
Lymphovascularinvasion	Positive	123 (22.8)	58 (22.6)	65 (26.3)	0.327
	Negative	381 (70.6)	199 (77.4)	182 (73.7)	
Perineural invasion	Positive	73 (13.9)	35 (13.7)	38 (15.5)	0.572
	Negative	427 (79.1)	220 (86.3)	207 (84.5)	
Tumor differentiation	Good	205 (38)	108 (46.4)	97 (40.9)	0.101
	Medium	252 (46.7)	122 (52.4)	130 (54.9)	
	Poor	13 (2.4)	3 (1.3)	10 (4.2)	
Tumor (T) stage	T1	16 (3)	10 (3.7)	6 (2.2)	0.130
	T2	85 (15.7)	48 (17.8)	37 (13.7)	
	T3	375 (69.4)	175 (64.8)	200 (74.1)	
	T4	64 (11.9)	37 (13.7)	27 (10)	
Lymph node(N) stage	N0	331 (61.3)	172 (63.7)	159 (58.9)	0.490
	N1	141 (26.1)	65 (24.1)	76 (28.1)	
	N2	68 (12.6)	33 (12.2)	35 (13)	
TNM stage	Stage-1	78 (14.4)	47 (17.4)	31 (11.5)	0.138
	Stage-2	253 (46.9)	124 (45.9)	129 (47.8)	
	Stage-3	209 (38.7)	99 (36.7)	110 (40.7)	
Adjuvant chemotherapy	Yes	352 (65.2)	170 (63)	182 (67.4)	0.278
	No	188 (34.8)	100 (37)	88 (32.6)	
Recurrence status	Yes	219 (40.6)	126 (46.7)	93 (34.4)	***0.004***
	No	321 (59.4)	144 (53.3)	177 (65.6)	
Latest status of patients	Alive	348 (64.4)	156 (58)	192 (71.6)	***0.001***
	Dead	189 (35)	113 (42)	76 (28.4)	
Albumin (g/L), median (min–max)		35 (17–50.8)	33 (17–48.2)	38.8 (21–50.8)	***<0.001***
ALP (U/L), median (min–max)		83.5 (29–396)	102 (59–396)	68.5 (29–109)	***<0.001***

AAPR: Albumin-to-alkaline phosphatase ratio, TNM: Tumor–Nodes–Metastases, ALP: alkaline phosphatase. * Chi-square, *t*-test or Kruskal–Wallis test. Statistically significant *p* values are written in bold-italic.

**Table 2:** Parameters significantly affecting overall survival in univariable Cox regression analysis, with a significant model created using these parameters in multivariable Cox regression.

		Univariable		Multivariable	
Variables		HR (95% CI)	*p* value	HR (95% CI)	*p* value
Lymphovascular invasion	Negative	Reference	**0.004**	Reference	0.150
	Positive	1.63 (1.16–2.28)		1.33 (0.90–1.96)	
Perineural invasion	Negative	Reference	**0.003**	Reference	0.544
	Positive	1.75 (1.21–2.52)		1.13 (0.75–1.79)	
TNM stage	Stage-1	Reference	**<0.001**	Reference	**0.002**
	Stage-2	1.80 (1.06–3.08)		1.15 (0.66–2.02)	
	Stage-3	2.77 (1.62–4.72)		2.09 (1.15–3.79)	
Recurrence status	No	Reference	**<0.001**	Reference	**<0.001**
	Yes	231.8 (57.5–934.6)		198.8 (49.1–805.7)	
Age of diagnosis	≤60	Reference	**<0.001**	Reference	**<0.001**
	>60	2.53 (1.87–3.42)		1.78 (1.29–2.46)	
Platelet count (10^3^/µL)		0.999 (0.997–1000)	**0.045**	1.000 (0.999–1.002)	0.516
Albumin–alkaline phosphatase ratio	High	Reference	**0.005**	Reference	**0.022**
	Low	1.50 (1.13–2.02)		1.45 (1.05–2.01)	
Neutrophil–lymphocyte ratio		1.023 (1.000–1.046)	0.053	-	-
Platelet–lymphocyte ratio		1.000 (0.999–1.001)	0.629	-	**-**

HR: Hazard ratio, CI: Confidence interval, TNM: Tumor–Nodes–Metastases. Statistically significant parameters are written in bold.

## Data Availability

The data presented in this study are available upon reasonable request from the corresponding author. The data are not publicly available due to ethical commitments for sensitive patient information.
